# Development of reverse phase protein microarrays for the validation of clusterin, a mid-abundant blood biomarker

**DOI:** 10.1186/1477-5956-7-15

**Published:** 2009-04-06

**Authors:** Adriana Aguilar-Mahecha, Christiane Cantin, Maureen O'Connor-McCourt, Andre Nantel, Mark Basik

**Affiliations:** 1 Lady Davis Institute for Biomedical Research, Department of Oncology, Montréal, Québec, H3T 1E2, Canada; 2 Biotechnology Research Institute, National Research Council of Canada, Montréal, Québec, H4P 2R2, Canada

## Abstract

**Background:**

Many putative disease blood biomarkers discovered in genomic and proteomic studies await validation in large clinically annotated cohorts of patient samples. ELISA assays require large quantities of precious blood samples and are not high-throughput. The reverse phase protein microarray platform has been developed for the high-throughput quantification of protein levels in small amounts of clinical samples.

**Results:**

In the present study we present the development of reverse-phase protein microarrays (RPPMs) for the measurement of clusterin, a mid-abundant blood biomarker. An experimental protocol was optimized for the printing of serum and plasma on RPPMs using epoxy coated microscope slides and a non-denaturing printing buffer. Using fluorescent-tagged secondary antibodies, we achieved the reproducible detection of clusterin in spotted serum and plasma and reached a limit of detection of 780 ng/mL. Validation studies using both spiked clusterin and clinical samples showed excellent correlations with ELISA measurements of clusterin.

**Conclusion:**

Serum and plasma spotted in the reverse phase array format allow for reliable and reproducible high-throughput validation of a mid-abundant blood biomarker such as clusterin.

## Background

The increasing application of genomics and proteomics technologies in medical research is making possible the development of "personalized medicine", i.e. medical care characterized by the use of biomarkers for the molecular diagnosis of different disease states and for the selection of therapies tailored to the individual's disease. Although both tissue and blood biomarkers are being discovered, blood derived-biomarkers are particularly attractive in the clinic since blood collection is inexpensive and relatively non-invasive and blood comes in contact with all tissues in the body. Unfortunately, the translation of putative blood biomarkers into clinical application has been hindered by the lack of a high-throughput technical platform for their validation. ELISAs are the standard method currently used for blood biomarker validation. However, the requirement for large sample volumes (~100 μl) and its low throughput make ELISA a costly method, both in time and biological material, not suited for the rapid validation of clinical samples.

A novel technology designed to measure protein levels in a high-throughput fashion is the protein microarray [1,2]. There are two main types of protein microarrays. The forward phase format consists of bait molecules, usually antibodies that are immobilized on the slide surface. Slides containing hundreds or thousands of antibodies are then incubated with the sample of interest (e.g., cell lysate or serum) allowing the simultaneous screening of a large number of putative protein biomarkers in a single test sample [1]. In the reverse phase array format, minute amounts of biological samples are printed onto the array surface. Cell and tissue lysates as well as biological fluids such as urine, CSF, serum and plasma can be spotted onto such arrays. Slides containing these sample sets are then incubated with antibodies targeted against a single protein of interest, one marker per slide. Analytes can be detected using amplification methods or with tagged secondary antibodies. Thus, reverse phase protein microarrays enable the high-throughput screening of thousands of clinical samples in a single microarray experiment [3].

Reverse phase protein microarrays (RPPMs) have been successfully applied for the profiling of post-translational modifications and cell signalling pathways in cell and tissue lysates [4-6] and the protocols for this application have been well described [7]. In contrast, only a few studies report the use of this type of array for serum and plasma applications with results limited to the detection of highly abundant proteins [8,9] using different protocols.

In the present study we describe the development of a protocol to print serum and plasma on RPPMs for the measurement of clusterin. Clusterin is a mid abundant blood protein present in serum and plasma in the μg/mL range [10]. Also known as apolipoprotein J, this apoptosis-related protein exists in two major isoforms. The nuclear form (nCLU), generated from an alternative splicing event, is a 55 kDa protein with pro-apoptotic properties [11]. The secreted form (sCLU) is a 75–80 kDa heavily glycosylated heterodimer composed of α and β chains of approx 34–37 kDa and has pro-survival functions [12]. Many recent studies have reported important roles of clusterin in carcinogenesis [13], tumorigenesis [14] and chemoresistance [15] and its expression level has been shown to be deregulated in several cancers, suggesting a potential clinical use as a cancer biomarker [13]. We have performed comparative analyses of clusterin detection using different slide chemistries and buffers for printing serum and plasma samples in the reverse phase format. We report the limit of detection, dynamic range, intra and inter array reproducibility as well as the results from validation studies using ELISAs with our optimized protocol.

## Methods

### Antibodies

The primary antibodies used were polyclonal anti-clusterin-α (C-18): sc-6419 (Santa Cruz, CA) and monoclonal anti-clusterin-α (B-5): sc-5289 (Santa Cruz, CA). Detection antibodies were Cy3 anti-mouse IgG and Cy3 anti-goat IgG (Jackson Immuno Research, U.S.A.). All primary antibodies were used at a 1:20 dilution in buffer (PBS containing 3% BSA) and all secondary antibodies were used at 1:100 dilution in the same buffer.

### Slides

Three types of slides were tested:

Nitrocellulose slides (Grace Bio-Labs, Bend, OR), MaxiSorp™ (Nunc Nalgene International) and SuperChip™ Glass Microarray slides with epoxyde surface coating (Erie Scientific company, Portsmouth, NH).

### Test Samples for Protocol Development and Optimization

Human plasma was obtained from Sigma and two-fold serial dilutions were prepared by adding 2× commercially available protein printing buffer (PPB) (ArrayIt^®^, TeleChem International, Sunnyvale CA) or Urea buffer (3 M Urea prepared in 1× PPB, 0.5% CHAPS, 32.5 mM DTT and 1% Pharmalyte 8-10-5). Control samples consisted of PPB or Urea buffer alone.

For proof of concept experiments, serial dilutions of mouse IgGs (Jackson Immuno Research, U.S.A.) and six serial dilutions of plasma (Sigma) were prepared in PPB. To assess the minimum difference detected and for ELISA validation studies, recombinant clusterin (produced in-house at the Biotechnology Research Institute) was prepared in series of two-fold dilutions in PPB. Two volumes of each dilution were added to corresponding tubes containing one volume of plasma (Sigma) and one volume of 2× PPB to obtain final clusterin concentrations ranging from 0.5 ng to 250 μg/mL and 0.9 to 500 μg/mL.

### General Protocol for Array Preparation

Diluted samples were loaded onto 384 well plates (Whatman), and then arrayed onto the different slides using a Virtek SDDC-2 arrayer (BioRad) and quill-type SMP3 contact pins (TeleChem, Sunnyvale, CA). The array chamber is equipped with a humidifier and humidity was set at 60% and temperature at 23°C during printing. Spot to spot distance was set to 400 microns and the average spot diameter obtained was 135–150 microns. After printing, slides were allowed to dry in the arrayer overnight before being directly used or kept in a dessicator until further processing.

Printed slides were blocked for 2 hours with PBS, 3% BSA. After blocking, slides were washed quickly in PBS, and incubated with primary antibody solutions for 90 min at room temperature. Slides were washed twice for 10 min in PBST (PBS containing 0.1% Tween 20) followed by one 5 min wash in PBS and were then incubated in solutions containing secondary antibodies conjugated to the fluorescent dye for 2 hours at room temperature in the dark. Slides were then washed again in PBST, as described above, and then rinsed briefly in deionized water to remove PBS buffer salts. The slides were air dried by spinning at 800 rpm for 5 min and scanned with a ScanArray Lite confocal fluorescent microarray scanner (Perkin Elmer).

### Data Analysis

Fluorescence was quantitated using the Histogram algorithm in QuantArray^® ^software (Packard Bioscience, Meridan, CT). The raw fluorescence units of each spot were background subtracted and the corrected fluorescence value was used to calculate the average fluorescence signal and standard deviation of replicate spots. For the analysis of replicate arrays we performed a scaled normalization to the median to minimize inter-array variability. Briefly, for a series of arrays we defined a normalization constant Ci by taking the median of the average fluorescent signals (average of replicate spots) of each array. We also defined a constant K by taking the median of the average fluorescent signals across all replicate arrays compared. We normalized all the arrays to the common total median intensity K by dividing all average fluorescent intensity readings from array i by Ci and multiplying by K. The median of all normalized values across arrays was calculated and considered the final fluorescent value of each sample.

For the analysis of clinical samples, because of the large number of data points, low intensity (raw fluorescence units < 1000) and defective spots were first eliminated for each array. Average, standard deviation and %CVs of replicate spots were calculated and a cut-off of 20% for acceptable reproducibility was set. For replicate spots with %CV > 20% either one outlier spot was eliminated if possible to bring the %CV below 20%, or the whole sample was eliminated from the analysis. Only reproducible data was included in the correlation with ELISAs and calculation of scaling factor (see below).

To compare RPPM data and ELISA data, we calculated a scaling factor by dividing the median intensity of each array Ci by the median ELISA value of all samples measured. The fluorescence intensity obtained in each spot on each array was then divided by the scaling factor to generate the scaled data.

Paired t-tests (GraphPad Prism Version 5) were performed on clinical samples to compare the different collection protocols. A value of p < 0.05 was considered as statistically significant.

### Western Blot Analysis

Monoclonal and polyclonal antibodies against clusterin were screened for specificity by Western Blot. The same human plasma that was used for protocol development was diluted 1/25, 1/50 and 1/100 in PBS and was incubated with denaturing buffer and boiled for 3 minutes. Denatured samples were loaded on a 10% SDS-PAGE and recombinant clusterin was used as a positive control. After gel migration (1 h at 150 V), proteins were transferred to a PDVF membrane. The membrane was incubated with 1:1000 goat anti-human clusterin antibody (Santa Cruz) as primary antibody and 1:10000 diluted HRP-conjugated donkey anti-mouse IgG as secondary antibody. After washing, the amount of bound HRP was visualized with the ECL method (GE Healthcare, UK).

### Clinical samples for Assay Validation

Blood was collected from eleven healthy non-fasting volunteers who participated in a pilot study analysing the effects of different blood collection protocols on plasma biomarker levels. This study was approved by the Research Ethics Office of the Jewish General Hospital. Informed consent was obtained for all volunteers participating in the study. Venipuncture was performed using a butterfly needle (22 G; Sarstedt) and blood was drawn from a sitting position. A total of five specimens per volunteer were collected in the following order: 2 × 4.5 ml Vacutainer™ CTAD tubes (BD Biosciences), 1× 10 mL serum tube with no additives (BD Biosciences) and 2 × 4.5 mL CTAD tubes. CTAD tubes were inverted 10 times to allow for adequate mixing of blood and buffer. For each pair of CTAD tubes, blood was pooled and 1.5 mL aliquoted in five 2 mL eppendorfs. One 1.5 mL aliquot was centrifuged immediately (time 0) at 1300 × g for 10 minutes at room temperature. The remaining four aliquots were left on the bench to be centrifuged 30 min, 60 min, 120 min and 24 hrs after time 0, respectively. Following centrifugation, approximately 500 μl of plasma was collected with a needle (16 G1 1/2; Sarstedt) and then filtered through an Acrodisc^® ^Syringe Filter unit (0.45 μm, 13 mm diameter) (Pall Life Sciences, MI) to obtain platelet poor plasma. Filtered plasma was aliquoted and immediately stored at -80°C.

For serum processing, the tubes were left to clot for 30 minutes and then centrifuged at 2000 rpm for 10 min at room temperature. Serum was collected leaving approximately 10% of serum above the buffy coat. To make it directly comparable to the CTAD protocol, serum was also filtered as mentioned above. Aliquots of 250 μL were made and stored immediately at -80°C (time 0) or left on the bench to be stored 30 min, 60 min, 120 min and 24 h after centrifugation.

All samples used in the present study had been thawed only once prior to this analysis. Our experience has shown no change in clusterin levels after five freeze/thaw cycles (data not shown).

### Validation using ELISA analysis

For the measurement of clusterin we used a Human Clusterin ELISA kit (BioVendor, Czech Republic). Samples were processed as recommended by the manufacturer and read in a FLUOstar OPTIMA microplate reader (BMG Labtech, Germany).

## Results

### Comparison of substrates and buffers

Important conditions for successful array preparation include good spot morphology, spot reproducibility and optimal protein attachment. Based on these criteria we initiated the development of protocols to print serum and plasma on reverse phase protein microarrays by testing combinations of substrates and buffers. We tested three different slide types, nitrocellulose, epoxy coated glass slides and MaxiSorp™ black polymer plastic slides. Test samples were prepared in two different buffers, a commercially available non-denaturing buffer (PPB), and a denaturing buffer (Urea). Test samples included five two-fold serial dilutions of human plasma. There were two subarrays per slide. The first subarray was composed of all test samples prepared in the Urea buffer spotted in triplicate, and the second subarray comprised samples prepared in PPB also spotted in triplicate.

**Figure 1:**
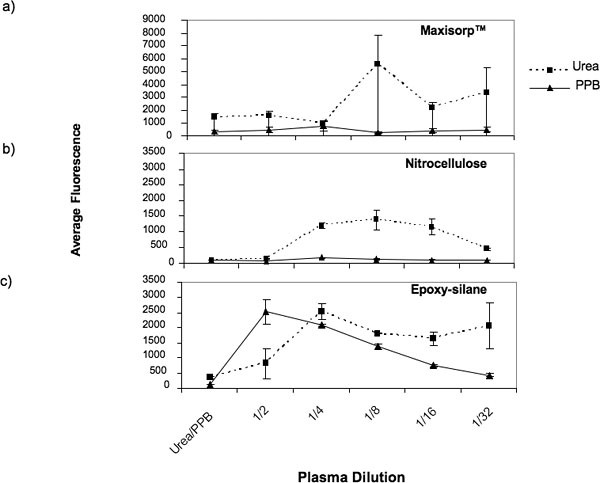
**Average fluorescence intensity of triplicate spots containing different plasma dilutions prepared in either PPB or Urea buffers and spotted onto (a) MaxisSorp™, (b) nitrocellulose and (c) epoxy coatedslides**. Each buffer was also spotted alone as negative controls on each slide.

Slides were incubated with a primary antibody against clusterin followed by Cy3 labeled secondary antibody. Results from this experiment are shown in Table 1 and Figure 1. Printing onto black plastic Maxisorp™ slides resulted in low fluorescence intensity for all samples printed with the PPB buffer. We observed some improvement in protein attachment with Urea, but the sample did not bind consistently on the Maxisorp™ slides resulting in very poor spot reproducibility and no concordance between the fluorescence intensities and the plasma dilutions (Figure 1a). Although nitrocellulose performed somewhat better with the Urea buffer, the conditions were not optimal for the attachment of concentrated plasma samples and higher background auto-fluorescence levels were observed (Figure 1b). Optimal results for all the criteria analysed were obtained when plasma samples were prepared in the non-denaturing buffer (PPB) and printed onto epoxy coated slides. Under these conditions, good linearity of the fluorescence intensity corresponding to the plasma serial dilutions was observed (Figure 1c). In addition, low background and the lowest spot to spot variability were also seen with this buffer and substrate combination. Epoxy coated slides together with the buffer PPB were used for all remaining experiments in this study.

**Table 1:** Criteria Analysed for different substrates and buffers

	Background		Spot Morphology		Spot Reproducibility	
Substrate/Buffer	PPB	UREA	PPB	UREA	PPB	UREA
Nitrocellulose	Moderate/High	Moderate/High	Squared	Circle/donuts	9.9	11.2
MaxiSorp™	Low	Low/Moderate	Squared	N/A	19.6	30.6
Epoxy	Low	Low/Moderate	Squared	N/A	6.7	15.7

### Limit of detection and dynamic range of clusterin detection

**Figure 2:**
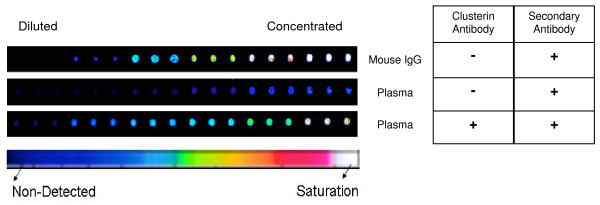
**Scanned image of fragments of RPPMs showing spotted triplicates of plasma and mouse IgGs probed with (+) or without (-) primary anti-clusterin antibody, and with secondary fluorescently labeled anti-IgG antibody**. Pseudo-color scale, dark blue to white corresponds to increasing fluorescence.

To ensure that the detection of endogenous clusterin was not due to non-specific binding from the secondary antibody, we printed two mini arrays each containing six serial dilutions of commercially available human plasma and six serial dilutions of mouse IgGs, each spotted in triplicate. One array was probed with a mouse primary antibody against human clusterin and a fluorescently labeled anti-mouse IgG secondary antibody. The second array was used as a negative control and was only incubated with the anti-mouse IgG secondary antibody. Figure 2 shows that a fluorescent signal corresponding to the detection of endogenous clusterin in plasma samples is only seen when both primary and secondary antibodies are used.

**Figure 3:**
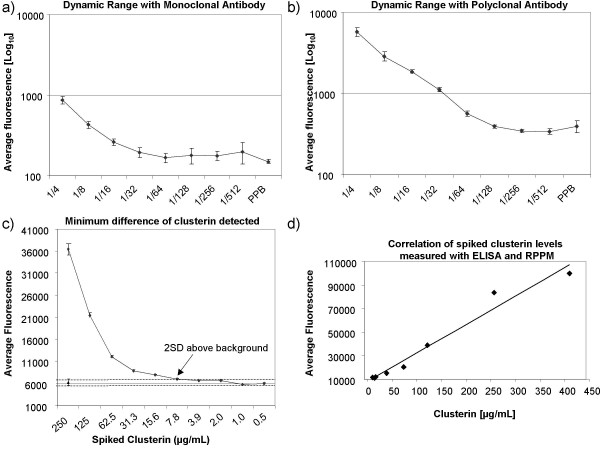
**Evaluation parameters of RPPMs**. (a) Detection of clusterin in plasma samples using monoclonal and (b) polyclonal primary anti-clusterin antibodies. The log_10 _average signal intensity for clusterin was plotted against plasma dilution 1/4 to 1/512. (c) Minimum difference in clusterin concentration detected on RPPMs. Twenty different concentrations of recombinant clusterin were spiked in plasma and the average fluorescence intensity of three slides median normalized was plotted against the ten highest clusterin concentrations. The average fluorescence level (▲) with the value of 2 standard deviations (---) for the ten lowest endogenous clusterin concentrations are used as background clusterin level in this experiment. Arrow points to the concentration of spiked clusterin that yielded a signal at least 2SD above background plasma clusterin. (d) Correlation of clusterin levels measured with ELISA and RPPM (r = 0.989). Clusterin was measured in plasma samples spiked with increasing concentrations (0.9–500 μg/ml) of recombinant clusterin.

Since different antibodies show different affinity and specificity for the same target protein, we compared a polyclonal and a monoclonal antibody against clusterin and assessed their limit of detection and dynamic range on reverse phase protein microarrays. First, the specificity of both antibodies was confirmed by western blot analysis using the same human plasma samples spotted onto the RPPM. The single band at ~37 kDa that is expected under reducing conditions and that corresponds to the cleaved alpha-chain of human sCLU was obtained for both the polyclonal antibody (see Additional file 1) and the monoclonal antibody (data not shown) confirming their specificity for clusterin. We prepared two slides where serial plasma dilutions were spotted in quadruplicate in two separate subarrays. Each array was probed with either the monoclonal or polyclonal antibody against clusterin. Quadruplicates of PPB were also spotted in each subarray to serve as negative controls. The background-subtracted mean fluorescent intensity of eight spots for each plasma dilution was plotted (Figure 3a and 3b). With both antibodies, the linearity of the signal for clusterin followed the plasma dilution series until the signal tapered and was not discernible from background level. With the polyclonal antibody, less variability was seen between spots and the fluorescent signal was more intense (10 fold difference) resulting in a dynamic range of 16 fold (plasma diluted 4 to 64 fold) compared to 8 fold (plasma diluted 4 to 32 fold) for the monoclonal antibody. Whole plasma and plasma diluted 2 fold were not spotted onto the array since our preliminary results had showed a lot of variability and signal saturation at these sample concentrations (data not shown). The limit of detection (LOD) was determined with a standard method making use of this dilution curve generated with the plasma sample of known concentration and identifying the amount of analyte that produces a fluorescent signal at least two standard deviations above the average fluorescence intensity of the background signal (eight control spots with PPB) [5,16]. Given an initial clusterin concentration of 50 μg/mL in the plasma samples used (measured with an ELISA kit) we estimate that the LOD reached with the polyclonal antibody is approximately 780 ng/mL. If we take into consideration the plasma dilution (plasma diluted 64 times) and the minute amount of sample per spot (estimated as 0.7 nL according to the manufacturer of the spotting pins) we can estimate that our RPPM can consistently detect at least 546 femtograms of clusterin per spot. Because of its superior results, the polyclonal anti-clusterin antibody was used for all remaining experiments.

### Minimum difference detected and reproducibility of RPPM

To identify the minimum difference in clusterin concentration that can be detected on our reverse phase microarrays, we prepared a 1:4 dilution of plasma in PPB and spiked it with increasing amounts of recombinant clusterin. Twenty concentrations of clusterin ranging from 0.5 ng to 250 μg per ml were spiked in a constant amount of diluted plasma and each sample was spotted in quadruplicate. Three arrays were probed thus resulting in a total of 12 data points for each concentration. Samples spiked with the ten lowest clusterin concentrations (250-0.5 ng/mL) yielded a low and relatively constant signal (data not shown). The average fluorescent signal obtained from these ten samples was attributed to endogenous clusterin present in the plasma sample. The minimum difference detected on our RPPMs was defined as the concentration of spiked clusterin yielding a fluorescent signal at least two standard deviations above the signal level for endogenous clusterin. We found that an increase of at least 8 μg/ml of clusterin can be reliably detected on our platform (Figure 3c). This is remarkable considering that the spiking was done in plasma diluted only four fold, conserving a fair degree of complexity compared to spiking done in buffer only. The minimum difference detected is well within the 50–250 μg/ml physiological range of plasma clusterin in humans.

One of the major challenges in protein microarray printing is to obtain consistent reproducibility between spots and between arrays. We analysed spot-to-spot variability and array-to-array variability with the results from this same experiment. For intra-slide spot reproducibility, we calculated the coefficient of variation (CV) of fluorescence intensity for each set of quadruplicate spots and analysed the distribution of %CVs in twenty sets of quadruplicates per array. Overall %CV values were below 10% for all of the samples, and the average %CV calculated for each array was 5.5%, 4.1% and 3.9% for the three arrays.

Inter-array reproducibility was assessed by averaging the fluorescence intensity of quadruplicates for each sample and comparing it across arrays, for which %CVs were calculated. We found that for replicate arrays the variability ranged from 10–16% with mean %CV of 13% when the raw average fluorescence data was used. In order to remove any systematic variation we applied a scaled to median normalization method to our data (see methods). Following normalization, the coefficient of variation between arrays was significantly reduced to a range of 0.1 – 8.4% with a mean %CV of 2.2%.

### Validation of RPPM results with ELISA

We validated the clusterin measurements performed on the reverse phase protein microarrays by comparing with results obtained from an ELISA assay performed on the same samples. Plasma diluted four fold was spiked with increasing concentrations of recombinant clusterin ranging from 0.9 μg/mL to 500 μg/mL. Each sample was measured in duplicate using a commercially available ELISA kit (BioVendor Inc) and in quadruplicate on a reverse phase microarray. Three subarrays were probed and the average fluorescence of 12 data points for each sample was plotted against its respective ELISA value. Figure 3d shows that results from both platforms correlate very well with a correlation coefficient r of 0.984. These results demonstrate that clusterin spiked in a complex plasma sample can be reliably measured using reverse phase protein microarrays.

### Clinical samples

**Figure 4:**
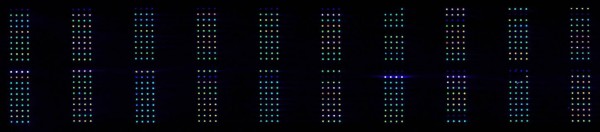
**Scanned image of slide containing 149 clinical samples spotted in quadruplicate and probed for clusterin**.

**Figure 5:**
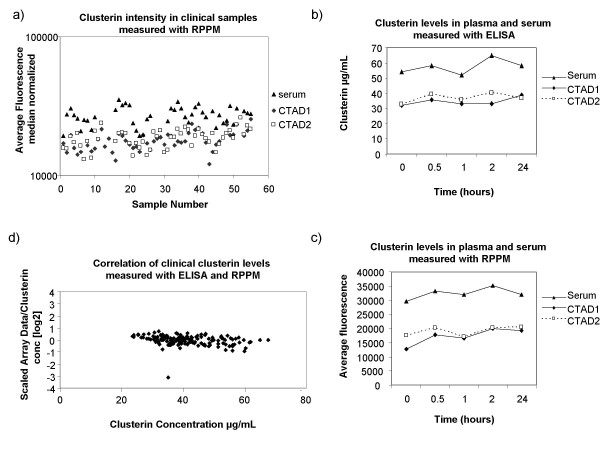
**Measurement of clinical samples using RPPMs andELISA**. (a) The log_10 _median clusterin intensity of all clinical samples analysed was plotted. (b) Clusterin levels in serum and CTAD plasma samples taken from the same individual and processed at different time points (0 min to 24 hrs) were measured with ELISA and (c) RPPM. (d) Correlation of RPPM data and ELISA values obtained for all clinical samples. The scaled data of all 16 data points per sample was averaged and then divided by its respective clusterin concentration measured by ELISA to obtain a ratio. For each sample, the log_2 _ratio was plotted against its respective clusterin concentration measured by ELISA.

In order to evaluate the clinical applicability of our platform, we used RPPMs to measure the levels of clusterin in 149 clinical blood samples. These clinical samples were obtained from eleven healthy individuals who participated in a pilot study to determine the effects of different blood processing protocols on levels of mid and low-abundant plasma biomarkers. As pre-analytical variability is one major factor of concern in proteomics studies, we used our platform to assess whether or not clusterin levels varied when using different blood processing protocols. For each subject, whole blood was collected in three blood collection tubes in the following order: citrate-theophylline-adenosine-dipyridamole (CTAD) tube, serum tube, and CTAD tube. We decided to use CTAD plasma collection tubes as they stabilize platelets and prevent platelet degranulation. Clusterin is present in significant quantities in platelets, and thus the use of different types of collection tubes could yield different clusterin concentrations from the same patient. Each blood sample was processed with a specific protocol testing the effect of different delays of blood processing after blood collection ranging from time zero to 24 hours. Plasma and serum samples were diluted 1:4 in buffer and spotted in quadruplicates, four replicate arrays were analysed resulting in 596 sets of quadruplicates or 2384 data points in total (Figure 4). In order to include only good quality array data in the validation analysis we filtered for spot quality (see methods). Overall spot reproducibility was very good with 84% of quadruplicate sets with %CV below 10%. For 18 sets the variability did not meet the criteria (%CV > 20%) and one spot was removed from the analysis in 15 of these quadruplicate sets. In total, 7 whole sets of quadruplicates were removed from the analysis due to low spot intensity or bad spot quality. All the remaining data for the four arrays was median normalized as previously described (methods), and for each sample, the median intensity of all 4 replicate arrays was considered to be the clusterin signal. The distribution of clusterin relative expression across the different samples analysed is shown in Figure 5a. We observed that the levels of clusterin detected on the protein microarray were significantly higher in serum samples when compared to CTAD (p < 0.001). On the other hand, delaying sample processing for up to 24 hours did not significantly affect clusterin serum and plasma levels (Fig 5b). Similar results were obtained after validation using an ELISA assay (Fig 5c). Since a much larger number of samples had been spotted compared to our previous tests, we again performed inter-array variability analysis and obtained very good reproducibility with an average %CV of 11%.

In order to directly compare the fluorescence intensities from reverse phase protein microarrays to the measured clusterin levels obtained by ELISA, we scaled the array data as reported by Janzi et al [9] and as described in the methods section. Briefly, for those samples with corresponding ELISA values (140 in total since no ELISA values were obtained for samples from one of the volunteers) a scaling factor was calculated and applied to each data point. The correlation between the RPPM data and the ELISA results for all samples is shown in figure 5d. Almost the totality of the samples analysed (98.6%) had a log_2 _ratio between 1 and -1 indicating a very good overall correlation across the range of clusterin concentrations.

Direct comparison of median normalized RPPM data to ELISA values was also performed. For each set of samples collected from the same subject a correlation to the corresponding ELISA values was performed. Therefore we obtained ten correlation coefficients with r values ranging from 0.51 to 0.93 and with median of 0.71.

## Discussion

The major bottleneck in translating newly discovered biomarkers to the clinic is the validation of these discoveries in controlled clinical samples [17]. We and others are developing a novel high-throughput platform to meet the challenge of biomarker validation in human blood samples, using reverse-phase protein microarrays (RPPMs). Although RPPMs for cell and tissue lysates are used extensively and protocols are available [18-20] there are few reports on the use of RPPMs for serum and plasma samples and no specific protocol has been defined [8,9,21,22]. In the present study we developed an optimal spotting protocol, including the choice of a printing buffer and surface chemistry, for the printing of plasma and serum in the reverse array format, and we assessed the applicability of RPPMs for the measurement of a mid-abundant plasma biomarker, clusterin.

The type of substrate and printing buffer used for microarray fabrication, in addition to sample properties such as viscosity and surface tension, have a major influence on array quality parameters including spot morphology, background and protein affinity binding [23]. In the preparation of reverse phase protein microarrays for cell and tissue lysates, nitrocellulose remains the most commonly used substrate [24-27]. This surface allows proteins to bind non-covalently (hydrophobic interaction) to the surface and also provides high binding capacity allowing the detection of very low abundant analytes when using catalysed signal amplification detection methods [5,18]. However, one of the drawbacks of nitrocellulose is its high intrinsic fluorescence that results in high background levels, thereby limiting the sensitivity of the assay when fluorescently labelled antibodies are used for detection [28]. In our hands, plasma binding to nitrocellulose was limited, and, together with the higher background levels, it resulted in a lower dynamic range for the measurement of an endogenous protein, clusterin, when compared to epoxy coated slides. Although nitrocellulose performs very well for printing denatured cell and tissue lysates, we did not find it yield satisfactory results for serum and plasma RPPMs when using a fluorimetric detection method compared to epoxy, therefore no further development to deal with the autofluorescence issue of nitrocellulose was pursued. We also found that MaxiSorp™ black polymer slides, which perform well in forward-phase antibody microarrays [29-31], showed the poorest binding results and spot morphology with both of the buffers tested on our reverse phase arrays, suggesting that plasma samples may be incompatible with the hydrophilic chemistry of the substrate. Our best results were obtained when epoxy coated slides and non-denaturing PPB buffer were used in combination. Epoxy surfaces offer several advantages including covalent immobilization, a more diffuse distribution of the proteins on the slide, and low non-specific background [32,33]. The more diffuse binding pattern of proteins on the slide may increase the chance of the antibody binding to its epitope since blood proteins are spotted in their native conformation with the PPB buffer.

In the present study we provide proof of principle for the detection of human endogenous clusterin, a mid-abundant blood protein, in plasma and serum using RPPMs and fluorescently labelled secondary antibodies. The sensitivity and specificity of any antibody based assay is largely dependent on antibody performance. We found that the polyclonal antibody targeted against clusterin showed a dynamic range 2-fold greater than that of the monoclonal antibody. Interestingly, spot variability was also reduced when slides were probed with the polyclonal antibody. It is worthwhile noting that both antibodies had demonstrated the pre-requisite specificity, recognizing a single band at the expected molecular weight in western blot analysis of the same complex sample used for the RPPM experiment. As with any use of reverse phase protein microarrays, careful screening of the specificity of antibodies is necessary; in addition, we suggest screening for dynamic range before selecting the optimal antibody to be used on the RPPM platform.

The sensitivity of any biomarker validation technology needs to be sufficiently high to enable the detection of low abundant protein biomarkers. Here we report a limit of detection (LOD) in the ng/mL range (780 ng/ml), which is approximately 100 fold less than the plasma levels of the endogenous mid abundant serum/plasma protein clusterin, using RPPMs in the absence of signal amplification detection methods. Although the LOD reached was essentially identical to that of the ELISA assay used in our validation studies (750 ng/mL), the sensitivity of our RPPM platform was significantly higher if we consider that we could detect this low amount of analyte from only 0.7 nL of biological sample. Our results suggest that the RPPM platform can be used for the high throughput and reproducible detection of mid abundant blood protein biomarkers. It is possible that the limit of detection can be lowered using different amplification techniques such as TSA amplification or the use of nanoparticles such as quantum dots. However, amplification methods were clearly not required for the accurate detection of clusterin on our platform.

We focused the development of our platform for the detection of clusterin. Blood clusterin levels have been shown to change in different disease states including numerous cancers [34] and systemic lupus erythematosus [35] as well as in diabetic type II patients and in patients with developing coronary heart disease, or myocardial infarction [36]. Preliminary evidence also suggests that measurement of clusterin in serum and plasma may be useful for the early detection of colorectal cancer [37] and for monitoring pVHL-defective renal carcinomas [38]. Moreover, changes in clusterin levels are being studied as surrogate biomarkers for treatment efficacy in clinical trials of anti-clusterin therapy in breast, lung and prostate cancer [39,40]. In our platform, the minimal difference of 8 μg of clusterin that can be detected between samples is clearly in the range of clinically relevant changes in plasma clusterin. Moreover, the good correlation with ELISA values and the low spot to spot variability and array to array variability observed when large numbers of clinical samples are screened further demonstrates that RPPMs for the detection of clusterin hold potential for clinical applicability. We feel that these results are all the more remarkable as they were obtained using only samples from healthy volunteers, in which clusterin levels showed very limited variation. In fact, our RPPM platform was useful to assess whether clusterin levels changed when using different blood processing protocols. We could observe a relationship between the levels of clusterin and the type of blood collection tube used. We used CTAD tubes which contain citrate-theophylline-adenosine-dipyridamole and inhibit platelet activation [41]. As clusterin is highly expressed in platelets [42], inhibiting platelet activation and thus the release of clusterin from platelets, is likely to explain the lower clusterin levels observed in CTAD plasma compared to serum. Our results are similar to those from a previous study which had recommended the use of citrate plasma for the measurement of clusterin [43], but this is, to our knowledge, the first report measuring plasma clusterin levels in CTAD tubes. Furthermore, the comparison of both CTAD specimens from the same individual showed that the levels of clusterin do not change from the first and third blood draw, thus suggesting that, when measuring clusterin, it may not be necessary to discard the first tube as sometimes recommended in blood collection guidelines. The results from our RPPM analysis and further validation with ELISA assays further confirm that blood collection protocols require careful standardization and that the differences in serum and plasma levels need to be taken into consideration in the course of clinical studies measuring clusterin levels.

Ideally, a clinically useful biomarker should be detected in serum or plasma. Prior to the clinical adoption of biomarker-based tests, putative biomarkers need to be rigorously validated in thousands of clinical samples. With RPPMs it is possible to spot hundreds if not thousands of serum and plasma samples on a single slide and analyze a biomarker of interest in one single experiment. Although the platform is presently limited by antibody availability and specificity, this is likely to change in the near future thanks to global initiatives such as the Human Antibody Initiative from HUPO and the Swedish Human Proteome Resource (HPR) program, which focus on the development of high quality antibodies against all human proteins [44].

One alternative method for biomarker validation is multiple reaction monitoring mass spectrometry (MRM-MS) [45]. This method is very attractive since it does not require antibodies for the detection and measurement of biomarkers; however, it has not yet reached the required sensitivity for low abundant proteins in complex samples such as serum and plasma. Moreover, although many biomarkers can be simultaneously monitored in a sample, it has a limited throughput since only one sample can be analyzed at a time. From a practical perspective, if an antibody is available for a biomarker of interest, antibody based methods such as RPPMs, are more easily adopted as validation tools than mass spectrometry based methods [46].

## Conclusion

In the present study we demonstrate the clinical applicability of RPPMs for the measurement of mid-abundant plasma proteins such as clusterin. Further technical improvements to the platform will have to be implemented to lower the limit of detection of plasma biomarkers to that of most clinically relevant markers, i.e. in the low nanogram/mL and picogram/mL range. With greater sensitivity, reverse phase protein microarrays offer the potential to speed up the validation process awaiting hundreds of putative disease biomarkers and their translation to the clinical setting.

## Abbreviations

PPB: Protein printing buffer; ELISA: Enzyme-linked immunosorbent assay; RPPM: Reverse-phase protein microarray; LOD: Limit of detection; CTAD: Citrate-theophylline-adenosine-dipyridamole; CV: Coefficient of variation; MRM-MS: multiple reaction monitoring mass spectrometry.

## Competing interests

The authors declare that they have no competing interests.

## Authors' contributions

AA analyzed and interpreted the microarray data, participated in collection of clinical samples, performed ELISA assays and drafted the manuscript, CC performed the microarray experiments and western blots, imaged and quantified the data, AN participated in the design of the study and helped in the analysis and interpretation of data. MO participated in the design of the study and in its coordination. MB conceived of the study, participated in its design and coordination and helped draft the manuscript. All authors read and approved the final manuscript.

## Supplementary Material

Additional file 1 **Western blot analysis of clusterin in human plasma samples**. it contains the results from the validation of the polyclonal antibody against clusterin screened for specificity by Western Blot. Click here for file
