# Supplementary figures and images for: Development of reverse phase protein microarrays for the validation of clusterin, a mid-abundant blood biomarker
Source: Proteome Sci. 2009 Apr 6;7:15. doi: 10.1186/1477-5956-7-15 (PMC2672067; doi:10.1186/1477-5956-7-15)

## Slide 1
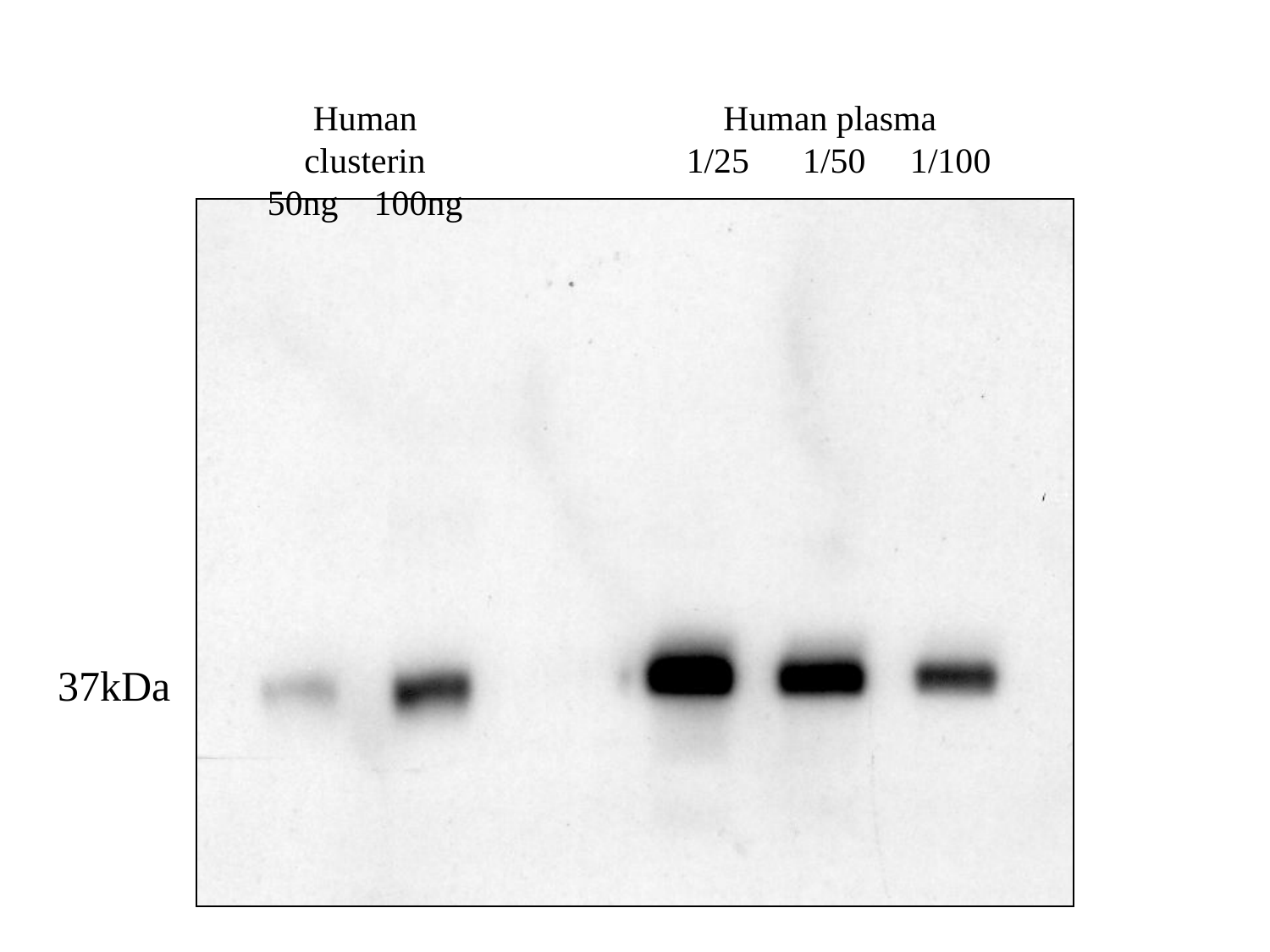

Human clusterin
50ng 100ng
Human plasma
 1/25 1/50 1/100
37kDa

Supplement: Additional file 1 — Western blot analysis of clusterin in human plasma samples. it contains the results from the validation of the polyclonal antibody against clusterin screened for specificity by Western Blot. [file 1477-5956-7-15-S1.ppt]
